# Comparison of apparent diffusion coefficient values in sentinel lymph nodes versus primary tumors for gastric cancer N staging

**DOI:** 10.3389/fonc.2025.1667430

**Published:** 2025-11-28

**Authors:** Liang-liang Yan, Jing Li, Jin-rong Qu, Hong-kai Zhang, He Zhang, Wei-hui Yu

**Affiliations:** 1 Department of Radiology, Affiliated Cancer Hospital of Zhengzhou University & Henan Cancer Hospital, Zhengzhou, China; 2 Department of Pathology, Affiliated Cancer Hospital of Zhengzhou University & Henan Cancer Hospital, Zhengzhou, China; 3 Department of Gastric Surgery, Affiliated Cancer Hospital of Zhengzhou University & Henan Cancer Hospital, Zhengzhou, China

**Keywords:** apparent diffusion coefficient, magnetic resonance imaging, sentinel lymph node, primary tumor, stomach neoplasms

## Abstract

**Purpose:**

To explore the differences in apparent diffusion coefficient (ADC) values based on the primary tumor and sentinel lymph node (SLN) for predicting N stages of gastric cancer (GC).

**Methods:**

One hundred and sixty histopathologically confirmed GC patients between April 2021 and October 2024 were prospectively recruited. Preoperative DW-MRI was performed, and ADC values from primary tumors (ADC_T_) and SLNs (ADC_LN_), along with their relative ratios (rADC_T_, rADC_LN_), were measured. Differences in these parameters across N stages were analyzed using the Kruskal-Wallis test. Receiver operating characteristic analysis was used to evaluate their diagnostic performances for predicting N0 vs. N1–3 stages, N0 + 1 vs. N2 + 3 stages, and N0 + 1 + 2 vs. N3 stages.

**Results:**

Significant differences were observed in ADC_T_, rADC_T_, ADC_LN_, and rADC_LN_ values across N stages (all *p* < 0.001). The AUC values of ADC_T_, rADC_T_, ADC_LN_, and rADC_LN_ for predicting N0 vs. N1 + 2 + 3 stages were 0.753, 0.727, 0.782, 0.792, respectively. The AUC values of ADC_T_, rADC_T_, ADC_LN_, and rADC_LN_ for predicting N0 + 1 vs. N2 + 3 stages were 0.776, 0.767, 0.844, 0.837, respectively. The AUC values of ADC_T_, rADC_T_, ADC_LN_, and rADC_LN_ for predicting N0 + 1 + 2 vs. N3 stages were 0.797, 0.792, 0.857, 0.848, respectively.

**Conclusions:**

Both primary tumor- and SLN-derived ADC values can effectively differentiate N stages among patients with GC. SLN-based ADC parameters exhibit superior diagnostic performance compared to primary tumor-based measurements in stratifying N-stage progression.

## Introduction

Gastric cancer (GC) has ranked fifth globally in both incidence and mortality as of 2022 (1). The TNM staging system serves as the cornerstone for GC assessment and treatment decisions in clinical practice (2). Importantly, N staging directly determines the extent of intraoperative lymph node dissection (3). Sentinel lymph nodes (SLNs), defined as the first nodes receiving tumor lymphatic drainage and serving as the initial metastatic site, may be crucial for N staging evaluation (4). Anatomically, SLNs typically reside in perigastric stations No. 1-6, and radiological criteria identify them as nodes with the largest short-axis diameter (5). The metastatic cascade involves tumor cell detachment from the primary lesion, invasion through high endothelial venules, and subsequent lymphatic dissemination (6). Given this mechanism, the status of SLNs may theoretically provide a more reliable indicator for N-stage evaluation than the primary tumor itself.

The apparent diffusion coefficient (ADC), derived from diffusion-weighted magnetic resonance imaging (DW-MRI), is one of the most robust quantitative parameters in functional imaging. ADC has demonstrated significant value in quantitatively assessing the N-stage of GC (7–13). Previous studies primarily focused on ADC values obtained from primary tumor regions for N-stage evaluation (8–13). Given the aforementioned metastatic cascade, ADC values obtained from SLNs might enable more accurate N-stage assessment. However, our comprehensive PubMed search identified no prior studies investigating the potential of SLN ADC values for N-staging. Thus, this study aims to determine whether ADC values from SLNs differ significantly from those of primary tumors in predicting the N-stage of GC.

## Materials and methods

### Patients

This prospective study was approved by the Institutional Review Committee of Affiliated Cancer Hospital, Zhengzhou University (IRB No. [2021]023). We recruited patients who underwent gastric DW-MRI at our institution between April 2021 and October 2024, and all participants provided informed consent. The inclusion criteria were: 1. Endoscopically biopsy-confirmed gastric adenocarcinoma; 2. Patients underwent gastric MRI including T1-weighted, T2-weighted, and DWI sequences. The exclusion criteria were: 1. Surgery was performed more than two weeks after the MRI examination; 2. SLNs (stations No. 1-6) with short-axis diameter <3 mm; 3. Discordance in size or spatial distribution between DWI findings and surgical pathology records; 4. Mucinous adenocarcinoma subtype; 5. Poor image quality or severe artifacts compromising accurate analysis.

### MRI technique

All MRI scans were performed using a 3.0T MR scanner (MAGNETOM Skyra, Siemens, Erlangen, Germany) with built-in 18-channel body and 32-channel spine coils. All patients were positioned supine in the head-first position. Patients fasted for 6–8 hours before MRI. Approximately 10 minutes prior to scanning, 10 mg anisodamine was administered intramuscularly. Patients were instructed to drink 800–1000 mL of water 1–2 minutes before the scan.

Scanning was performed according to standard MRI protocols, with specific sequence scan parameters as follows: 1. T1WI: TR/TE = 4.34 ms/2.68 ms, slice thickness = 3 mm, FOV = 380 mm × 380 mm; 2. T2WI: TR/TE = 4000–8000 ms/96 ms, slice thickness = 3 mm, FOV = 380 mm × 380 mm; 3. DWI: TR/TE = 2600 ms/51 ms, slice thickness = 3 mm, FOV = 340 mm × 340 mm, *b* = 50, 800 s/mm^2^(A low b-value of 50 s/mm² helps to mitigate T2 shine-through effects, enabling better differentiation of true diffusion restriction from T2-prolongation); 4. DCE: TR/TE = 3.87 ms/1.82 ms; section thickness = 2.5 mm; FOV = 380; flip angle = 12°.

### Image interpretation

**Figure 1:**
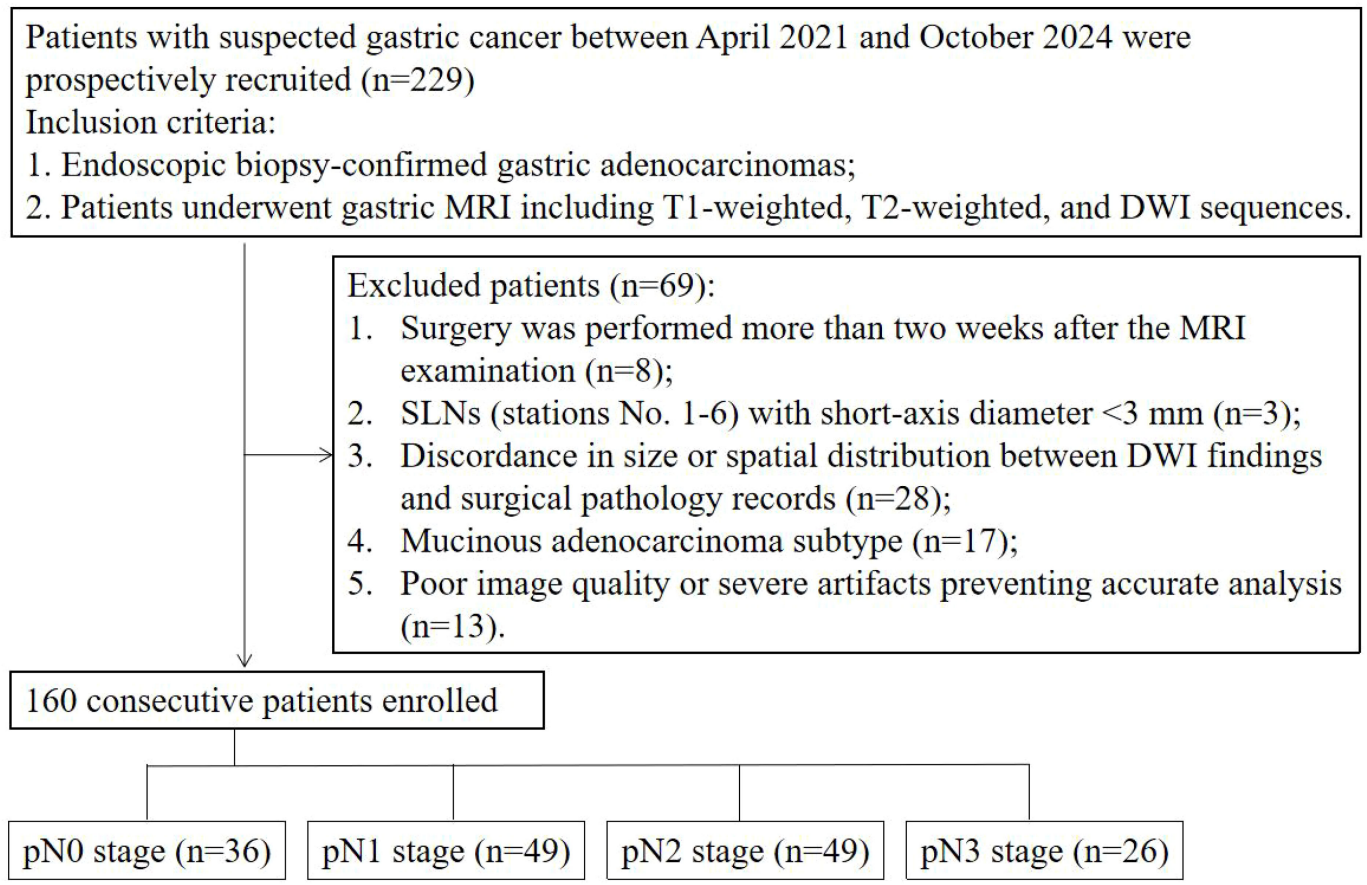
Flow diagram of the study patients. SLN, sentinel lymph node.

**Figure 2:**
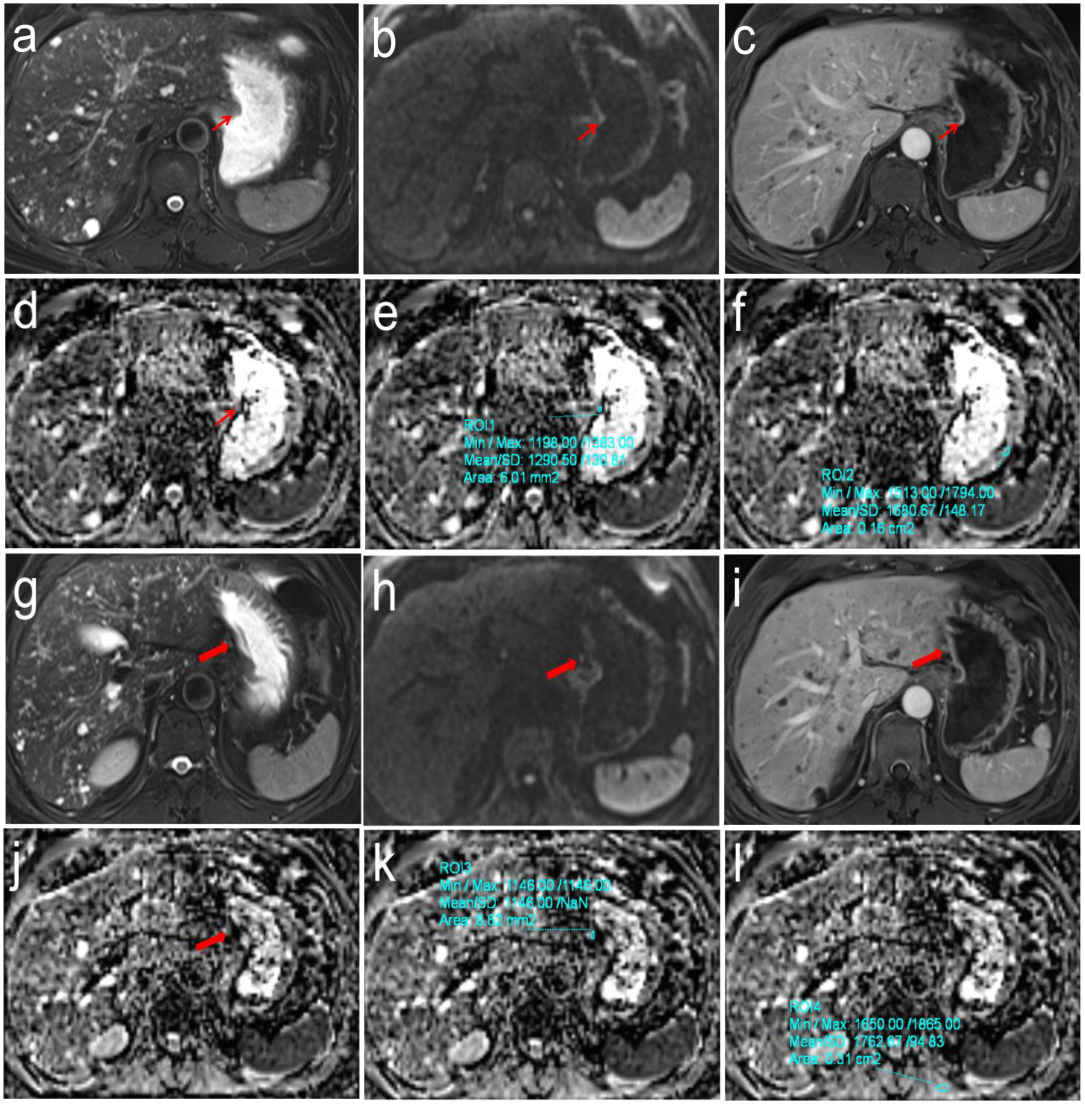
Pathologically confirmed gastric corpus adenocarcinoma (pT1N0M0) in a 62-year-old male. **(a-f)** T2WI, DWI and DCE images showed the location of primary tumor (Thin red arrow). The ADC values of primary tumor and normal gastric wall of the same axial section were 1.291 × 10^-3^mm2/s and 1.681 × 10^-3^mm2/s, respectively, the correspond rADCT value were 0.768; **(g-l)** T2WI, DWI and DCE images showed the location of perigastric sentinel lymph nodes (No. 3) (Thick red arrow). The short-axis diameter was about 3.2mm. The ADC values of target sentinel lymph nodes and the left vertical spinal muscle at the same axial section were 1.146 × 10^-3^ mm2/s and 1.763 × 10^-3^ mm2/s, respectively, the correspond rADCLN value were 0.650.

The DW-MRI data were transferred to the SyngoVia post-processing workstation. All data were independently analyzed by two radiologists with 7 and 11 years of work experience without knowing the pathological N staging results. First, the primary tumor and SLN (stations No. 1-6) were identified using T2WI, DWI, and DCE images. For each case, only the SLN with the largest short-axis diameter was selected for analysis. The ADC values of primary tumor (ADC_T_) and SLN (ADC_LN_) were measured on the corresponding ADC maps by manually drawing a freehand ROI covering the solid component of tumor with necrotic areas avoided. Reference ADC (rADC_T_ and rADC_LN_) values were obtained from ADC_T_ value/normal gastric wall ADC value of the same axial section, ADC_LN_ value/left erector spinae muscle ADC value of the same axial section, respectively (**Figures 1**, **2**).

### Determination of pathologic N stage

All resected specimens, including both gastric primary tumors and SLNs, were systematically grouped, labeled with their respective anatomical locations and sizes, and subsequently submitted to the pathology department for standard formalin fixation, paraffin embedding, sectioning, and HE staining. The pathological evaluation was conducted using the American Joint Committee on Cancer/Union for International Cancer Control (AJCC/UICC) 8th edition as the evaluation criteria. The criteria for determining SLN metastasis were lymph node short-axis diameter greater than 10mm or microscopically determined cancer cell infiltration. The evaluation criteria of N stage were: N0 stage, no regional lymph node metastasis; N1 stage, 1–2 regional lymph node metastases; N2 stage, 3–6 regional lymph node metastases; N3 stage, more than 7 regional lymph node metastases.

### Statistical analysis

All statistical analysis were performed using the SPSS 22.0 (IBM Corp., Armonk, NY, USA) and MedCalc 20.218 (MedCalc Software Ltd, Ostend, Belgium). The measured ADC_T_, rADC_T_, ADC_LN_, and rADC_LN_ values were expressed as mean ± standard deviation. Intraclass correlation coefficient (ICC) test was performed to determine the consistency of ADC_T_, rADC_T_, ADC_LN_, and rADC_LN_ values measured by two radiologists using Bland-Altman analysis. The ICC between 0.00 and 0.20 was defined as poor correlation; 0.21-0.40 as fair correlation; 0.41–0.60 as moderate correlation; 0.61–0.80 as good correlation; and 0.81–1.00 as excellent correlation. The Kruskal Wallis test was used to compare differences in ADC_T_, rADC_T_, ADC_LN_, and rADC_LN_ values between different N stages of GC. Receiver operating characteristic (ROC) curves were used to determine the diagnostic performance for predicting N0 vs. N1 + 2 + 3 stages, N0 + 1 vs. N2 + 3 stages, and N0 + 1 + 2 vs. N3 stages. The area under the curve (AUC) value of 0.85–1 was defined as good diagnostic performance, 0.70-0.84 as moderate diagnostic performance, and 0.50-0.69 as poor diagnostic performance. Pairwise comparisons between different AUCs were performed using the DeLong test. *p* < 0.05 was considered statistically significant.

## Results

### Study population

**Figure 3:**
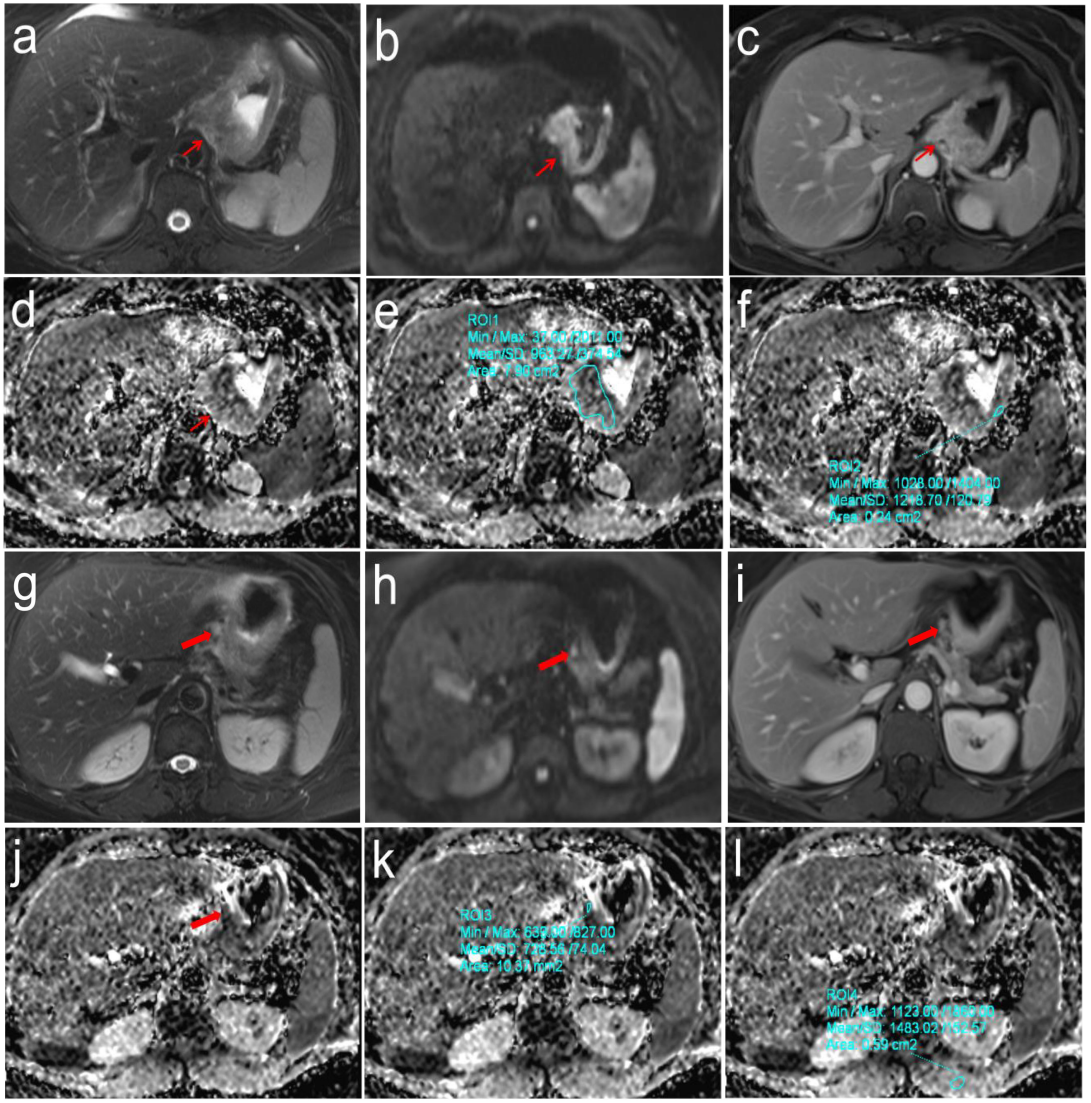
Pathologically confirmed gastric cardia adenocarcinoma (pT3N2M0) in a 57-year-old female. **(a-f)** T2WI, DWI and DCE images showed the location of primary tumor (Thin red arrow). The ADC values of primary tumor and normal gastric wall of the same axial section were 0.963 × 10^-3^mm2/s and 1.219 × 10^-3^mm2/s, respectively, the correspond rADCT value were 0.790; **(g-l)** T2WI, DWI and DCE images showed the location of perigastric sentinel lymph nodes (No. 2) (Thick red arrow). The short-axis diameter was about 6.2mm. The ADC values of target sentinel lymph nodes and the left vertical spinal muscle at the same axial section were 0.729 × 10^-3^ mm2/s and 1.483 × 10^-3^ mm2/s, respectively, the correspond rADCLN value were 0.492.

A total of 229 cases were initially collected. Among these, 8 cases were excluded because surgery was performed more than two weeks after the MRI examination; 3 cases were excluded due to SLNs (stations No. 1–6) with a short-axis diameter <3 mm; 28 cases were excluded because of discordant SLN findings between DWI and surgical pathology (anatomical station mismatch or >5 mm short-axis diameter discrepancy); 17 cases were mucinous adenocarcinoma; and 13 cases had poor image quality or severe artifacts that precluded accurate analysis. Ultimately, 160 cases (mean age 60.9 ± 9.7 years; 128 men) were included in this study. The flowchart of the included cases is shown in **Figure 3**.

The clinical pathological characteristics of this study are shown in **Table 1**. Age, gender, tumor location, tumor thickness, short-axis diameter of selected gastric lymph nodes, pathological TNM staging, the number of selected gastric lymph nodes, and lymph node dissection were recorded. Among them, tumor thickness, short-axis diameter of selected gastric lymph nodes, pathological T stage, pathological TNM staging, lymph node dissection demonstrated statistically significant differences in distinguishing the four N-stage groups (all *p* < 0.01).

**Table 1:** The clinicopathological characteristics of GC.

Clinicopathological characteristics	Total (n=160)	N0 (n=36)	N1 (n=49)	N2 (n=49)	N3 (n=26)	χ^2^ value	*P*-value
Age (years, mean ± SD)	60.9 ± 9.7	61.2 ± 9.2	62.4 ± 9.3	59.8 ± 10.1	60.9 ± 9.7	1.983	0.576
Gender						4.531	0.210
Male	128	29	40	42	17		
Female	32	7	9	7	9		
Tumour location						5.272	0.153
Cardia	93	15	34	28	16		
Corpus	35	11	4	13	7		
antrum	32	10	11	8	3		
Tumor thickness (mm, mean ± SD)	14.7 ± 6.5	9.4 ± 4.6	13.6 ± 5.2	17.3 ± 4.5	19.7 ± 8.1	51.382	< 0.001
Short-axis diameter of selected lymph nodes (mm, mean ± SD)	11.0 ± 5.9	4.3 ± 0.7	10.4 ± 4.2	13.1 ± 4.0	17.3 ± 6.1	95.000	< 0.001
pT-stage*						54.081	< 0.001
T1	5	5	0	0	0		
T2	37	18	17	1	0		
T3	118	13	32	48	26		
T4a	0	0	0	0	0		
pM-stage*						–	–
M0	160	36	49	49	26		
M1	0	0	0	0	0		
pTNM stage*						113.470	< 0.001
Stage I	23	23	0	0	0		
Stage II	34	13	19	1	1		
Stage III	103	0	30	48	25		
Stage IV	0	0	0	0	0		
Number of selected gastric lymph nodes						0.616	0.893
No. 1 (right cardia)	10	3	5	2	0		
No. 2 (left cardia)	50	9	15	17	9		
No. 3 (lesser curvature)	73	19	20	23	11		
No. 4 (greater curvature)	3	0	0	0	3		
No. 5 (suprapyloric)	10	2	3	4	1		
No. 6 (infrapyloric)	14	3	6	3	2		
Lymph node dissection						129.627	< 0.001
D1	94	36	49	9	0		
D2	55	0	0	40	15		
D3	11	0	0	0	11		

*According to AJCC / UICC TNM Staging of GC (8th Edition), *GC* Gastric cancer.

### Interobserver agreement

**Figure 4:**
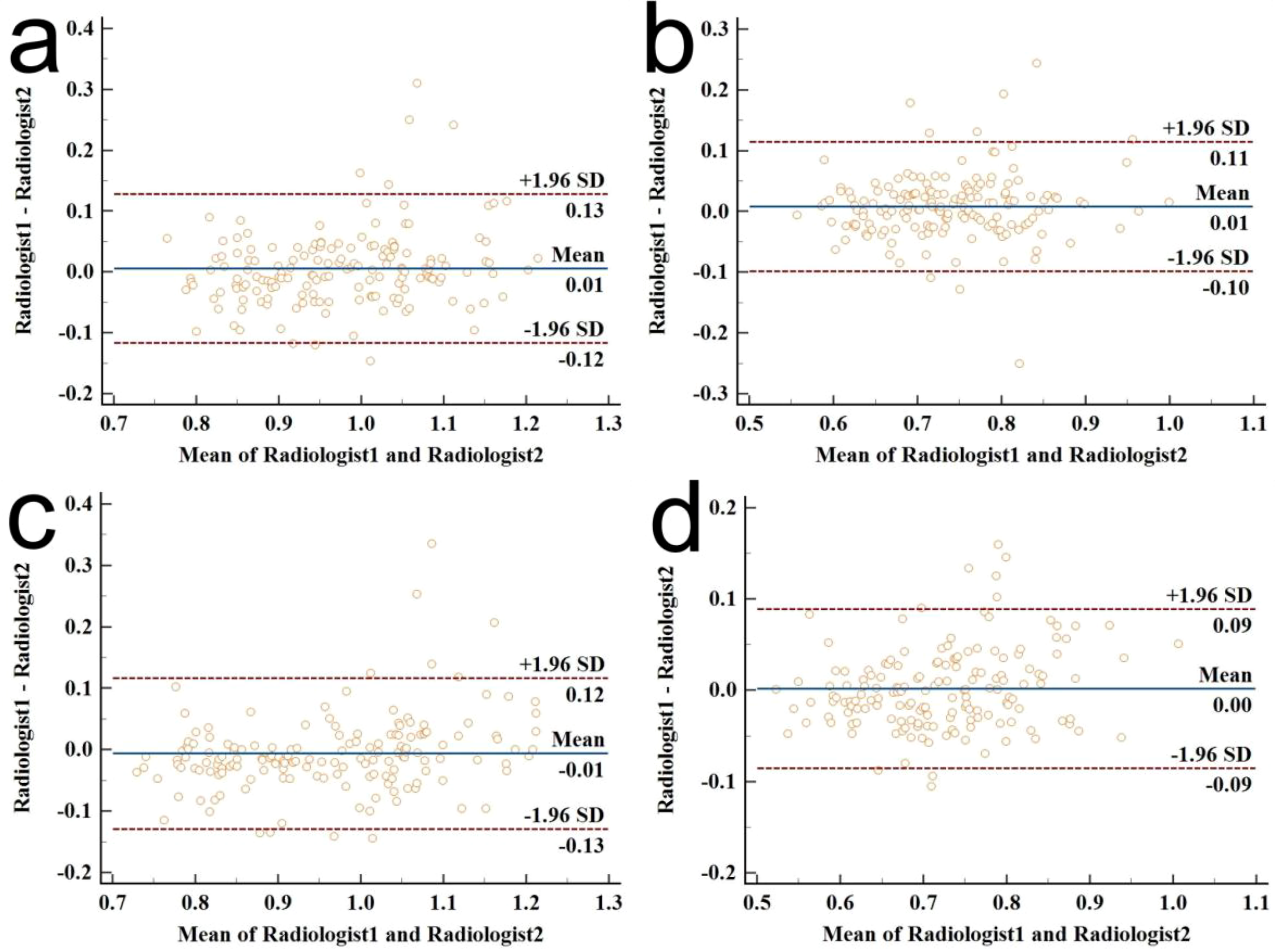
Bland–Altman plot diagrams for the interobserver agreement of ADC value measurements between the two radiologists. **(a)** ICC for ADCT measurements; **(b)** ICC for rADCT measurements; **(c)** ICC for ADCLN measurements; **(d)** ICC for rADCLN measurements.

The consistency analysis of ADC_T_, rADC_T_, ADC_LN_, and rADC_LN_ values measured by two radiologists is shown in **Appendix Table 1**; **Figure 4**. The ICC values ranged 0.813-0.890, indicating an excellent correlation.

### ADC parameters analysis

**Table 2** showed that ADC_T_, rADC_T_, ADC_LN_, rADC_LN_ have statistically significant differences in distinguishing different N stages of GC (all *p* < 0.001). The ADC_T_, rADC_T_, ADC_LN_, and rADC_LN_ values were negative correlation with N stage.

**Table 2:** Differences analysis of different ADC values between N stages.

N stage	ADC_T_ (×10^-3^mm^2^/s)	rADC_T_	ADC_LN_ (×10^-3^mm^2^/s)	rADC_LN_
N0 stage (n=36)	1.04 ± 0.09	0.79 ± 0.08	1.06 ± 0.09	0.79 ± 0.07
N1 stage (n=49)	1.00 ± 0.08	0.75 ± 0.08	1.01 ± 0.08	0.75 ± 0.08
N2 stage (n=49)	0.94 ± 0.09	0.71 ± 0.08	0.91 ± 0.10	0.69 ± 0.07
N3 stage (n=26)	0.89 ± 0.09	0.66 ± 0.05	0.85 ± 0.09	0.64 ± 0.05
χ² value	43.445	40.132	63.863	58.069
*p*-value	<0.001	<0.001	<0.001	<0.001

The data was expressed as mean ± standard deviation, *ADC* Apparent diffusion coefficient.

**Figure 5:**
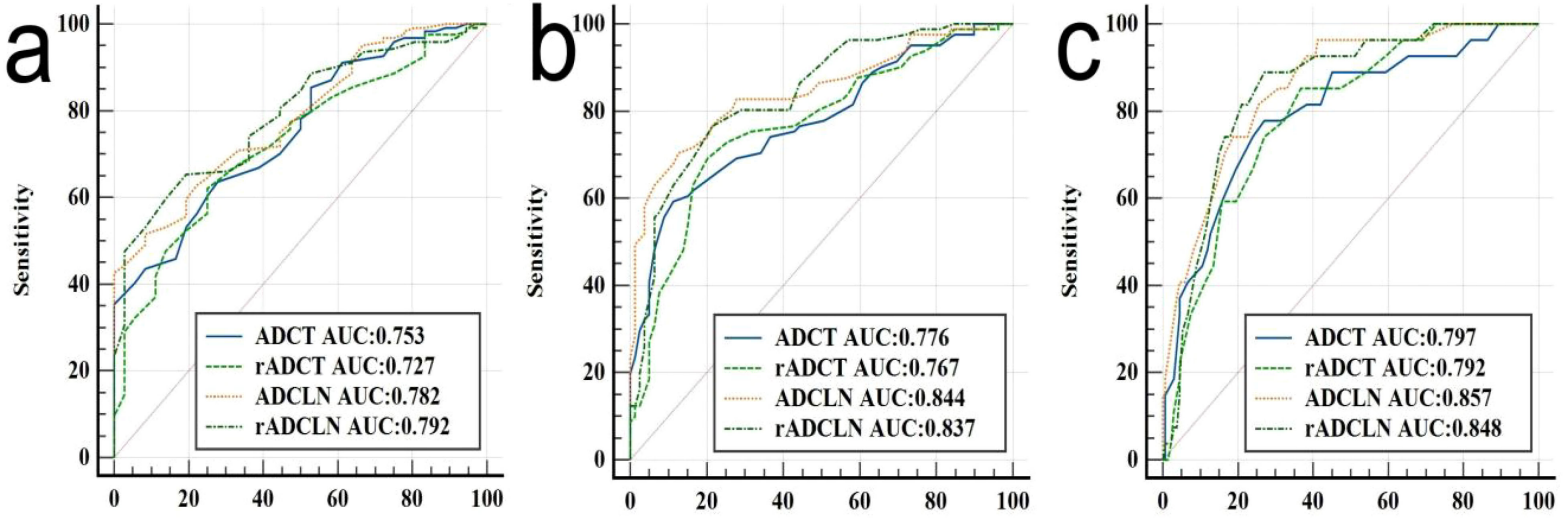
ROC analysis of different ADC for predicting **(a)** N0 vs. N1 + 2 + 3 stages, or **(b)** N0 + 1 vs. N2 + 3 stages, and **(c)** N0 + 1 + 2 vs. N3 stages of gastric cancer.

The ROC curve indicated that the AUC values of ADC_T_, rADC_T_, ADC_LN_, and rADC_LN_ for predicting N0 vs. N1 + 2 + 3 stages were 0.753, 0.727, 0.782, 0.792, respectively (**Table 3**, **Figure 5a**). In the pairwise comparison of ROC curves, there were statistically significant differences in AUC values between rADC_T_ and rADC_LN_ (Delong test, Z = 2.381, *p* = 0.017), while there was no statistically significant difference between other pairwise comparisons (all *p* > 0.05) (**Table 4**).

**Table 3:** ROC analysis of different ADC for predicting N0 vs. N1+2+3 stages of GC.

Parameter	AUC (95% CI)	*p-*value	Cut off value	Sensitivity (%)	Specificity (%)	PPV (%)	NPV (%)
ADC_T_ (×10^-3^mm^2^/s)	0.753 (0.678~0.817)	< 0.001	1.10	96.0	25.0	80.1	66.7
rADC_T_	0.727 (0.651~0.794)	< 0.001	0.88	97.6	16.7	38.0	92.6
ADC_LN_ (×10^-3^mm^2^/s)	0.782 (0.788~0.904)	< 0.001	1.10	95.2	33.3	83.1	66.7
rADC_LN_	0.792 (0.796~0.910)	< 0.001	0.82	93.6	33.3	82.9	60.0

*ADC*, Apparent diffusion coefficient; *AUC*, Area under the curve; *CI* ,Confidence interval; *PPV*, Positive predictive value; *NPV* , Negative predictive value.

**Table 4:** Pairwise comparisons between different AUCs.

Parameter	N0 vs. N1 + 2 + 3 stages		N0 + 1 vs. N2 + 3 stages		N0 + 1 + 2 and N3 stages	
	Z	*p-*value	Z	*p-*value	Z	*p-*value
ADC_T_ vs. rADC_T_	0.596	0.551	0.262	0.794	0.106	0.915
ADC_T_ vs. ADC_LN_	1.222	0.222	3.162	0.002	2.215	0.027
ADC_T_ vs. rADC_LN_	1.001	0.317	1.919	0.055	1.128	0.260
rADC_T_ vs. ADC_LN_	1.162	0.245	2.065	0.039	1.647	0.100
rADC_T_ vs. rADC_LN_	2.381	0.017	2.934	0.003	2.153	0.031
ADC_LN_ vs. rADC_LN_	0.272	0.786	0.259	0.796	0.315	0.752

*ADC*, Apparent diffusion coefficient; *AUC*, Area under the curve.

The AUC values of ADC_T_, rADC_T_, ADC_LN_, and rADC_LN_ for predicting N0 + 1 vs. N2 + 3 stages were 0.776, 0.767, 0.844, 0.837, respectively (**Table 5**, **Figure 5b**). In the pairwise comparison of ROC curves, there were statistically significant differences in AUC values between ADC_T_ and ADC_LN_, rADC_T_ and ADC_LN_, rADC_T_ and rADC_LN_ (Z = 3.162, 2.065, 2.934, *p* = 0.002, 0.039, 0.003, respectively), while there was no statistically significant difference between other pairwise comparisons (all *p* > 0.05) (**Table 4**).

**Table 5:** ROC analysis of different ADC for predicting N0 + 1 vs. N2 + 3 stages of GC.

Parameter	AUC (95% CI)	*p-*value	Cut off value	Sensitivity (%)	Specificity (%)	PPV (%)	NPV (%)
ADC_T_ (×10^-3^mm^2^/s)	0.776 (0.704~0.838)	<0.001	0.92	59.3	88.6	84.2	68.0
rADC_T_	0.767 (0.694~0.830)	<0.001	0.71	69.1	79.8	778.4	71.6
ADC_LN_ (×10^-3^mm^2^/s)	0.844 (0.779~0.897)	<0.001	0.93	70.4	87.3	85.1	74.2
rADC_LN_	0.837 (0.771~0.891)	<0.001	0.71	76.5	78.5	78.5	76.5

*ADC*, Apparent diffusion coefficient; *AUC*, Area under the curve; *CI*, Confidence interval; *PPV*, Positive predictive value; *NPV*, Negative predictive value.

The AUC values of ADC_T_, rADC_T_, ADC_LN_, and rADC_LN_ for predicting N0 + 1 + 2 vs. N3 stages were 0.797, 0.792, 0.857, 0.848, respectively (**Table 6**, **Figure 5c**). In the pairwise comparison of ROC curves, there were statistically significant differences in AUC values between ADC_T_ and ADC_LN_, rADC_T_ and rADC_LN_ (Z = 2.215, 2.153, *p* = 0.027, 0.031, respectively), while there was no statistically significant difference between other pairwise comparisons (all *p* > 0.05) (**Table 4**).

**Table 6:** ROC analysis of different ADC for predicting N0 + 1 + 2 vs. N3 stages of GC.

Parameter	AUC (95% CI)	*p-*value	Cut off value	Sensitivity (%)	Specificity (%)	PPV (%)	NPV (%)
ADC_T_ (×10^-3^mm^2^/s)	0.797 (0.726~0.856)	<0.001	0.84	37.0	95.5	62.5	88.2
rADC_T_	0.792 (0.721~0.852)	<0.001	0.62	22.2	95.5	50.0	85.8
ADC_LN_ (×10^-3^mm^2^/s)	0.857 (0.793~0.908)	<0.001	0.80	40.7	95.5	64.7	88.8
rADC_LN_	0.848 (0.783~0.900)	<0.001	0.60	29.6	94.7	53.3	86.9

*ADC*, Apparent diffusion coefficient; *AUC*, Area under the curve; *CI*, Confidence interval; *PPV*, Positive predictive value; *NPV*, Negative predictive value.

## Discussion

This prospective study investigated the diagnostic performances of ADC and rADC values measured in primary tumors and SLNs for N-stage classification in GC. Quantitative analysis demonstrated that both tumor-based ADC and SLN-based ADC parameters effectively discriminated between different N stages. Notably, SLN-based ADC parameters exhibited significantly higher diagnostic performance than tumor-based ADC parameters for predicting N0 vs. N1 + 2 + 3 stages, or N0 + 1 vs. N2 + 3 stages, and N0 + 1 + 2 vs. N3 stages.

Our findings demonstrated that ADC_T_ can effectively differentiate N stages in GC, corroborating previous reports in the literature (9–11). The underlying physiological basis lies in how ADC values quantify water molecule diffusion. With advancing N stage, more aggressive tumor proliferation leads to higher cellular density and reduced extracellular space. These changes restrict water molecule mobility, thereby leading to the observed progressive decrease in ADC values (14).

This study demonstrated that both ADC_LN_ and rADC_LN_ values could effectively differentiate between N stages in GC. To our knowledge, no prior studies have investigated N staging using lymph node-based ADC parameters. This gap in the literature may be attributed to several technical challenges: 1. Difficulties in achieving precise radiological-pathological correlation for individual lymph nodes; 2. A high necrosis rate in metastatic lymph nodes, which can introduce bias into ADC measurements; 3. The presence of multiple metastatic lymph nodes in advanced GC, complicating the selection of nodes for measurement; 4. Interference from severe chemical shift artifacts in certain lymph nodes. To mitigate these limitations, our study employed stringent selection criteria by focusing on SLNs, thereby improving measurement accuracy and minimizing potential errors.

Our findings demonstrate the superior diagnostic performance of SLN-based over tumor-based ADC values in differentiating N0 vs. N1 + 2 + 3 stages, N0 + 1 vs. N2 + 3 stages, and N0 + 1 + 2 vs. N3 stages in GC. We propose that the shorter disease duration of SLN metastases compared to the primary tumor underlies this difference. Given the marked propensity of adenocarcinoma for necrosis (15), the more established primary tumor is likely to harbor more necrotic foci. Since necrosis elevates ADC values by reducing diffusion restriction (16)—and because our ROIs, despite stringent protocols, might have included microscopic necrosis—ADC_T_ measurements could be systematically inflated. This would diminish their utility for staging. In contrast, the greater cellular integrity of SLN metastases maintains more consistent diffusion restrictions, which better reflect the metastatic burden.

We introduced rADC measurements with the original intention of normalizing values to mitigate inter-scanner variability. However, this study revealed that rADC—whether derived from primary tumors or SLNs—demonstrated either inferior or comparable diagnostic performance compared to absolute ADC values across all three N-stage subgroups. DeLong’s test further confirmed that the differences in AUC between rADC and ADC were not statistically significant. Consequently, selecting absolute ADC values as the standard parameter for N-stage stratification proves more clinically practical than relative ADC, as it eliminates the need for additional measurements of normal gastric wall or reference muscle ADC values, thereby facilitating broader clinical adoption.

Our research demonstrates that ADC_LN_ effectively distinguishes between N0 and ≥N1 stages in GC, consistent with previous findings (17–20). This capability has important implications for clinical decision-making regarding lymph node dissection. Current guidelines recommend D1 lymphadenectomy for N0 stage patients, while D2 lymphadenectomy is indicated for ≥N1 stage cases (3, 21). Furthermore, this study provides a novel perspective for predicting patient survival by differentiating between N0 + 1 and N2 + 3 stages. Clinically, cases with metastasis confined only to the SLNs are mostly classified as N1 disease. As reported by Jeong et al. (22), the 5-year survival rate for patients with SLN metastasis only was 73.1%, compared to merely 39.6% for those with distant lymph node metastasis. Our study demonstrates that ADC_LN_ (optimal cutoff: 0.93 × 10^-^³ mm²/s) may be used to predict patient prognostic outcomes.

The literature indicates that lymph nodes with a short-axis diameter exceeding 5 mm in GC exhibit a significantly increased likelihood of metastasis (23). In our study, the short-axis diameters of selected SLNs in the N1-N3 stages consistently exceeded 5 mm, aligning with these findings. In addition, to evaluate the impact of ADC values on N0 staging, we included SLNs with short axis diameters ranging from 3–5 mm as the research subjects.

This study excluded cases with skip lymph node metastasis, as prior studies reported a skip metastasis rate of 3.9%–5.3% in GC cases (24–26). Additionally, establishing standardized assessment criteria for skip metastatic lymph nodes presents inherent challenges.

Due to mucinous adenocarcinoma characteristically high extracellular mucin content (27)—which results in imaging features and ADC measurements that differ significantly from non-mucinous GCs—and its relatively low incidence (2.8-6.6% of cases (28)), we excluded mucinous adenocarcinoma cases from our final analysis.

This study has several limitations. First, it is a single-center study, which may introduce some bias into the results. Second, we did not evaluate the correlation with T stage or overall clinical staging; this will be the focus of future research. Third, the current first-line recommendations for GC TNM staging are endoscopic ultrasound and CT, while MRI is only a second-line recommendation (2). This suggests that the widespread adoption of our findings remains challenging. However, we note that MRI has gradually been recognized as a first-line recommended examination in expert consensus on TNM staging for other gastrointestinal tumors, such as esophageal and rectal cancer.

## Conclusion

To sum up, both ADC values and rADC values based on primary tumors and the SLNs can be used to distinguish N stage of GC, and SLN-based ADC values exhibit superior diagnostic performance for predicting N0 vs. N1 + 2 + 3 stages, N0 + 1 vs. N2 + 3 stages, and N0 + 1 + 2 vs. N3 stages of GC.

## Data Availability

The raw data supporting the conclusions of this article will be made available by the authors, without undue reservation.

## Supplementary material

Supplementary Table 1 Consistency analysis of different ADC values measured by two radiologists. The data was expressed as mean ± standard deviation; *ICC*, Intraclass correlation coefficient; *CI*, Confidence interval; *ADC*, Apparent diffusion coefficient.
