# Supplementary material for: Comparison of apparent diffusion coefficient values in sentinel lymph nodes versus primary tumors for gastric cancer N staging
Source: Front Oncol. 2025 Nov 28;15:1667430. doi: 10.3389/fonc.2025.1667430 (PMC12698429; doi:10.3389/fonc.2025.1667430)
Supplement: Supplementary Table 1 — Consistency analysis of different ADC values measured by two radiologists. The data was expressed as mean ± standard deviation; ICC, Intraclass correlation coefficient; CI, Confidence interval; ADC, Apparent diffusion coefficient. [file Table1.docx]

**APPENDIX TABLE 1 |** Consistency analysis of different ADC values measured by two radiologists

|  | Radiologist 1 | Radiologist 2 | ICC (95% CI) | *p*-value |
| --- | --- | --- | --- | --- |
| ADC_T_ (×10^-3^mm^2^/s) | 0.98 ± 0.11 | 0.97 ± 0.10 | 0.831 (0.776~0.874) | <0.001 |
| rADC_T_ | 0.74 ± 0.09 | 0.73 ± 0.09 | 0.813 (0.753~0.860) | <0.001 |
| ADC_LN_ (×10^-3^mm^2^/s) | 0.96 ± 0.14 | 0.97 ± 0.12 | 0.881 (0.841~0.911) | <0.001 |
| rADC_LN_ | 0.72 ± 0.10 | 0.72 ± 0.09 | 0.890 (0.852~0.918) | <0.001 |

The data was expressed as mean ± standard deviation; *ICC* Intraclass correlation coefficient, *CI* Confidence interval, *ADC* Apparent diffusion coefficient
